# Supplementary material for: Video based monitoring systems for hand hygiene compliance auditing: What do patients think?
Source: PLoS One. 2023 Mar 9;18(3):e0281895. doi: 10.1371/journal.pone.0281895 (PMC9997901; doi:10.1371/journal.pone.0281895)
Supplement: S1 File — (DOCX) [file pone.0281895.s001.docx]

|  | |  | **Susan Wakil School of Nursing and Midwifery  Faculty of Medicine and Health**  **University of Sydney** |
| --- | --- | --- | --- |
|  |  | |  |
|  | **CHIEF INVESTIGATOR (SUPERVISOR)**  **Professor Ramon Z. Shaban**  ***Clinical Chair of Infection Prevention and Control*** | | Room 0.4.20  Westmead Institute for Medical Research  The University of Sydney  NSW 2006 AUSTRALIA  Telephone: +61 2 8627 3117  Email: [ramon.shaban@sydney.edu.au](mailto:ramon.shaban@sydney.edu.au)  Web: <http://www.sydney.edu.au/> |

Video Based Electronic Surveillance for Hand Hygiene Auditing: An exploratory and evaluative study

**PARTICIPANT INFORMATION STATEMENT**

1. **What is this study about?**

You are invited to take part in a research study about a hybrid approach hand hygiene auditing that incorporates video based electronic surveillance.

Hand hygiene is an essential aspect of patient safety and hence auditing of healthcare worker practice is required in all public healthcare facilities in Australia under the auspices of the National Hand Hygiene Initiative. Data is collected by trained auditors using direct observation and while this method is considered the “gold standard” is it resource intensive in terms of auditor training, auditing time and data entry requirements. Furthermore, there are questions as to the potential impacts that the presence of an auditor may have on the behaviour of the healthcare workers who are being observed (known as the Hawthorne effect). In an attempt to improve auditing efficacy and accuracy of auditing, various electronic methods have been proposed however, none have been compatible with Australian data collection requirements.

Phase 1 of this study developed specifications for a hybrid direct observation/electronic surveillance method of hand hygiene auditing including the camera based technology for hand hygiene auditing .

Phase 2 of the study was a trial of concept in simulation to assess the efficacy, accuracy and ease of use of the technology.

Phase 3 explores the attitudes to healthcare workers and patients to the potential use of the system developed in phase 1 and trialled in phase 2.

As a healthcare consumer/patient you have been invited to participate in this part of the study because you expressed an interest in exploring further the issues raised when you completed the online survey. This Participant Information Statement tells you about the research study. Knowing what is involved will help you decide if you want to take part in the research. Please read this sheet carefully and ask questions about anything that you don’t understand or want to know more about.

Participation in this research study is voluntary.

By giving your consent to take part in this study you are telling us that you:

- Understand what you have read.
- Agree to take part in the research study as outlined below.
- Agree to the use of your personal information as described.

You will be given a copy of this Participant Information Statement to keep.

1. **Who is running the study?**

The study is being carried out by the following researchers:

- **Professor Ramon Z. Shaban**

Principal Investigator & Supervisor

University of Sydney and Western Sydney Local Health District

- **Ms Katherine J. McKay**

Principal Investigator & Student Researcher

University of Sydney & Eastern Health

- **Dr. Patricia E. Ferguson**

Principal Investigator Associate Supervisor

University of Sydney

Ms Katherine J. McKay is conducting this study as the basis for the degree of Doctor of Philosophy at The University of Sydney. This will take place under the supervision of Professor Ramon Z. Shaban and Dr Patricia E Ferguson.

1. **What will the study involve for me?**

You are invited to participate in a brief interview.

The interview would held at a time and place that is convenient and comfortable you. It is anticipated that the interview will take approximately 30 - 60 minutes or less and will discuss your thoughts and feelings about both the current method of direct human observational auditing and the developed and trialled video based electronic surveillance model. We are seeking to understand what you see as both the positives and negatives of such an approach as well as things that would “make it work” and things that would “get in the way”.

For the purpose of this research, this interview will be audio recorded. Audio recordings will be transcribed by researchers and will have names omitted to ensure confidentiality. These recordings will remain in a secure filing cabinet within the infection prevention and control service, Eastern Health, Victoria. Should you wish to, a copy of the interview transcript will be available for you to review.

All recordings will be stored in a secure filing cabinet within the infection prevention and control service, Eastern Health, Victoria. Should you wish to, a copy of the transcript will be available for you to review.

1. **How much of my time will the study take?**

Approximately 30 to 60 minutes

1. **Who can take part in the study?**

Health care consumers/patients or their families/carers are encouraged to participate.

1. **Do I have to be in the study? Can I withdraw from the study once I've started?**

Being in this study is completely voluntary and you do not have to take part. Your decision whether to participate will not affect your current or future relationship with the researchers or anyone else at the University of Sydney.

If you decide to take part in the study and then change your mind later, you are free to withdraw at any time. You can do this by contacting any of the above listed researchers*.*

You may also refuse to answer any questions that you do not wish to answer during the interview.

1. **Are there any risks or costs associated with being in the study?**

Aside from giving up your time, we do not expect that there will be any risks or costs associated with taking part in this study.

1. **Are there any benefits associated with being in the study?**

We cannot guarantee that you will receive any direct benefits from being in the study. The benefits that may arise through your participation in this project are mostly altruistic in nature and may arise from knowing that you are advancing knowledge regarding how practice changes may be introduced in an effective and cost efficient way.

1. **What will happen to information about me that is collected during the study?**

By providing your consent, you are agreeing to us collecting personal information about you for the purposes of this research study. Your information will only be used for the purposes outlined in this Participant Information Statement, unless you consent otherwise.

Audio recording of the interviews will be transcribed and all names and other identifying information will be removed to ensure confidentiality. Anonymised transcripts will be stored in secure filing cabinet within the Infection Prevention and Control Service at Eastern Health. Information will be retained for a period of 7 years, after which the information will be destroyed using confidential paper disposal service. Digital audio recordings will destroyed by deleting recorded information. Completed consent forms will be kept in a locked filing cabinet within the Infection Prevention and Control Service at Eastern Health. Any information obtained in connection with this research project that can identify you will remain confidential and will only be used for the purpose of this research project. It will only be disclosed with your permission, except as required by law.

In any publication and/or presentation, information will be provided in such a way that you cannot be identified, except with your permission. Unique identifiers will be allocated as a means of ensuring confidentiality and anonymity

1. **Can I tell other people about the study?**

Yes, you are welcome to tell other people about the study.

1. **What if I would like further information about the study?**

When you have read this information, Ms Katherine McKay will be available to discuss it with you further and answer any questions you may have. If you would like to know more at any stage during the study, please feel free to contact any of the researchers

- Principal Investigator and Supervisor **Professor Ramon Z. Shaban**

Email: [ramon.shaban@sydney.edu.au](mailto:ramon.shaban@sydney.edu.au)

Phone: +61 (0)2 8627 3117

- Principal Investigator and Student Researcher **Ms Katherine McKay**

Email: [kmck4637@uni.sydney.edu.au](mailto:kmck4637@uni.sydney.edu.au)

Phone: 0404809496

- Principal Investigator and Associate Supervisor **Doctor Patricia Ferguson**

Email: [Patricia.Ferguson@health.nsw.gov.au](mailto:Patricia.Ferguson@health.nsw.gov.au)

1. **Will I be told the results of the study?**

You have a right to receive feedback about the overall results of this study. You can tell us that you wish to receive feedback by ticking the box on the participant consent for and providing your contact details or by contacting any of the above listed researchers. This feedback will be in the form of a brief 1-2 page summary. You may also request copies of any publications arising from this study in the same manner. You will receive this feedback after the study is finished.

1. **What if I have a complaint or any concerns about the study?**

Research involving humans in Australia is reviewed by an independent group of people called a Human Research Ethics Committee (HREC). The ethical aspects of this study have been approved by the HREC of the University of Sydney **2019/387** As part of this process, we have agreed to carry out the study according to the *National Statement on Ethical Conduct in Human Research (2007).* This statement has been developed to protect people who agree to take part in research studies.

If you are concerned about the way this study is being conducted or you wish to make a complaint to someone independent from the study, please contact the university using the details outlined below. Please quote the study title and protocol number.

The Manager, Ethics Administration, University of Sydney:

- - **Telephone:** +61 2 8627 8176
  - **Email:** [human.ethics@sydney.edu.au](mailto:human.ethics@sydney.edu.au)
  - **Fax:** +61 2 8627 8177 (Facsimile)

# This information sheet is for you to keep

|  | |  | **Susan Wakil School of Nursing and Midwifery Faculty of Medicine and Health**  **University of Sydney** |
| --- | --- | --- | --- |
|  | ABN 15 211 513 464 | |  |
|  | **CHIEF INVESTIGATOR (SUPERVISOR)**  **Professor Ramon Z. Shaban**  ***Clinical Chair of Infection Prevention and Control*** | | Room 0.4.20  Westmead Institute for Medical Research  The University of Sydney  NSW 2006 AUSTRALIA  Telephone: +61 2 8627 3117  Email: [ramon.shaban@sydney.edu.au](mailto:ramon.shaban@sydney.edu.au)  Web: <http://www.sydney.edu.au/> |

**Video Based Electronic Surveillance for Hand Hygiene Auditing: An exploratory and evaluative study**

**PARTICIPANT CONSENT FORM**

I, ................................................................................... [PRINT NAME], agree to take part in this research study.

In giving my consent I state that:

- I understand the purpose of the study, what I will be asked to do, and any risks/benefits involved.
- I have read the Participant Information Statement and have been able to discuss my involvement in the study with the researchers if I wished to do so.
- The researchers have answered any questions that I had about the study and I am happy with the answers.
- I understand that being in this study is completely voluntary and I do not have to take part. My decision whether to be in the study will not affect my relationship with the researchers or anyone else at the University of Sydney or Eastern Health now or in the future.
- I understand that I can withdraw from the study at any time.
- I understand that I may stop the interview at any time if I do not wish to continue, and that unless I indicate otherwise any recordings will then be erased and the information provided will not be included in the study. I also understand that I may refuse to answer any questions I don’t wish to answer.
- I understand that personal information about me that is collected over the course of this project will be stored securely and will only be used for purposes that I have agreed to. I understand that information about me will only be told to others with my permission, except as required by law.
- I understand that the results of this study may be published, and that publications will not contain my name or any identifiable information about me.

I consent to:

- **Audio-recording** YES NO
- **Being contacted about future studies** YES NO

**I would like to review my interview transcripts** YES NO

**I would like to receive feedback about the overall results of this study** YES NO

If you answered **YES**, please indicate your preferred form of feedback and address:

Postal: _______________________________________________________

___________________________________________________

Email: ___________________________________________________

...................................................................

**Signature**

....................................................

**PRINT name**

..................................................................................

**Date**
