# Supplementary material for: Video based monitoring systems for hand hygiene compliance auditing: What do patients think?
Source: PLoS One. 2023 Mar 9;18(3):e0281895. doi: 10.1371/journal.pone.0281895 (PMC9997901; doi:10.1371/journal.pone.0281895)
Supplement: S2 File — (DOCX) [file pone.0281895.s002.docx]

# Interview Question Guide – Healthcare Consumers

**Non-identifiable demographic questions**

1. Have you used a healthcare service in Australia within the last 12 months?
2. Approximately how many times in the last 12 months have you or a family member/friends accessed a healthcare service?
3. Which state or territory healthcare service do you mostly access?
4. What type of healthcare service did you or your friend/relative use or visit most recently?

EG

- Metropolitan public hospital
- Metropolitan private hospital
- Regional public hospital
- Regional private hospital
- Ambulatory/outpatient service
- Community clinic service
- Other

1. How do you describe your current gender identity?
2. What is your age?

| Description of Direct Observational Hand Hygiene Auditing:  The current method of collecting information about hand hygiene compliance is called direct observation. It involves a trained hand hygiene auditor being present in the ward or treatment area, watching the healthcare workers providing care and indicating when they clean their hands. The auditor works out if the hand hygiene is done at the correct time to best protect the patient from infection, according to the World Health Organisation criteria. The auditor may be quite obvious, or discrete. |
| --- |

| **Share screen to show video of Direct Observational auditing**  **Video 1** |
| --- |

- Have you had experience of direct observational (human) auditing taking place whist you or your relative were a consumer of health care?
- (if yes) Did you receive an explanation about the process of auditing?
- How did you feel about this? (if no experience … “how do you think you would feel about this process?”)
- Do you think this is a good method of assessing healthcare workers’ practice?
- Why/why not?
- Do you feel that this method would change the HCWs behaviours – in what way?
- Do you feel that this method is an invasion of the patient’s privacy – why/why not?

| Video-based monitoring system:  Video based monitoring systems would involve the placement of cameras in the patient care area (e.g. in the patient’s room)  These cameras may record continuously or turn on only in the presence of a healthcare worker via a proximity tag or in response to movement or sounds.  Cameras would record HCW-patient interactions which would then be audited for hand hygiene compliance.  The following “rules” would apply to this auditing method:   - Patients would be informed of the auditing process and have the chance to opt out completely or intermittently – that is, to have the camera turned off for the entire time or during certain care activities. - Footage would be kept on secure hospital servers for a maximum of 48-hours and then be automatically deleted. - Auditing of hand hygiene compliance would be performed from the footage in a confidential manner by specially trained auditors – the footage would only be viewed by the designated auditor or a member of the Infection Prevention service. - Footage would not be utilised for any other purposes than those described, and would not be retained or released except as required by law.   Major or critical incidents (eg, criminal activities, patient or staff assault) would trigger a review process as per normal hospital policy and procedure. |
| --- |

| **SHOW SAMPLE FOOTAGE RECORDED WITH VIDEO MONITORING SYSTEMS FOR THE PURPOSE OF AUDING HAND HYGIENE PRACTICES**  **Video 2**  **Video 3**  **Video 4** |
| --- |

- What is your first reaction to this idea of using video cameras to record healthcare worker practice so that it can be audited for quality/compliance?
- Are you able to explain why you feel this way?
- Are you aware of the use of cameras is other aspects of daily life (eg CCTV) – how do you feel about these uses?
- What do you think would be good about this approach to hand hygiene auditing?
- What problems or issues do you see with this approach; do you have any concerns about it?
- Do you think that the use of cameras would be an invasion of patient privacy?
- It is anticipated that this approach has the potential to improve patient safety through better HCW practice and hence better quality of care – does this balance/mitigate the potential for invasion of privacy ?
- Is there any modification/caveats/rules/guarantees that would be needed to make this system more acceptable? This might include technical or operational features
  - Possible prompts ….
    - Camera’s which activate only when the HCW is in the room
    - A light or other indicator that tuns on when the filming is occurring
    - The patient can turn the camera off when they want to
    - No recording of audio
    - Camera’s positioned so that the view is from above and/or behind not face on
    - Automatic facial pixilation
- Are there any other “safety” aspects of the system that you think patients would need to be accepting of the system
- Are there any other “safety” aspects of the system that you think HCWs would need to be accepting of the system
- It was stated that major incidents would trigger a review as per normal hospital policy
  - What kind of things would you consider “major Incidents”
  - What should this review process involve?
- If this approach was to be used do you think consent would be required? If so, how should it be obtained?
  - E.g. possible prompts
    - Standard/routine practice that occurs unless a patient says they don’t want it
    - Signs advising that recording may take place
    - Verbal explanation and consent
    - Written consent
- It is anticipated that footage would be deleted within 48 hours to 7 days of recording – how do you feel about this?
- The potential to use cameras for multiple purposes has been raised… how do you feel about other uses, can you make any suggestions
- Possible prompts:
  - Staff safety/assault prevention
  - Falls prevention
  - Suicide watch
  - Telehealth
  - Family conferences/video chats
- Do you have any other thoughts about this proposed approach?
