# Supplementary material for: Video based monitoring systems for hand hygiene compliance auditing: What do patients think?
Source: PLoS One. 2023 Mar 9;18(3):e0281895. doi: 10.1371/journal.pone.0281895 (PMC9997901; doi:10.1371/journal.pone.0281895)
Supplement: Attachment — Submitted filename: Response to reviewers_VMS_McKay.docx [file pone.0281895.s003.docx]

| Journal Requirements:  When submitting your revision, we need you to address these additional requirements. |  |
| --- | --- |
| 1. Please ensure that your manuscript meets PLOS ONE's style requirements, including those for file naming. The PLOS ONE style templates can be found at  [https://journals.plos.org/plosone/s/file?id=wjVg/PLOSOne_formatting_sample_main_body.pdf](https://protect-au.mimecast.com/s/zuWWC4QOPEiJpl4MXIW7aNZ?domain=journals.plos.org) and  [https://journals.plos.org/plosone/s/file?id=ba62/PLOSOne_formatting_sample_title_authors_affiliations.pdf](https://protect-au.mimecast.com/s/fwr2C5QPXJi0V6k5BFxUMcc?domain=journals.plos.org). | Thank you – this has been amended |
| 2. Please provide additional details regarding participant consent. In the ethics statement in the Methods and online submission information, please ensure that you have specified (1) whether consent was informed and (2) what type you obtained (for instance, written or verbal, and if verbal, how it was documented and witnessed). If your study included minors, state whether you obtained consent from parents or guardians. If the need for consent was waived by the ethics committee, please include this information. | Thank you – this has been amended and a copy of the PICF is added as a supporting file. Please see also study setting and participants section which details that participant age range was 19 to 85 years |
| If you are reporting a retrospective study of medical records or archived samples, please ensure that you have discussed whether all data were fully anonymized before you accessed them and/or whether the IRB or ethics committee waived the requirement for informed consent. If patients provided informed written consent to have data from their medical records used in research, please include this information | Not applicable |
| Once you have amended this/these statement(s) in the Methods section of the manuscript, please add the same text to the “Ethics Statement” field of the submission form (via “Edit Submission”). | Thank you |
| For additional information about PLOS ONE ethical requirements for human subjects research, please refer to [http://journals.plos.org/plosone/s/submission-guidelines#loc-human-subjects-research](https://protect-au.mimecast.com/s/hWTiC6XQ4LfonyLj5fL-kbT?domain=journals.plos.org). | Not applicable |
| 3. We note that table 1 includes an image of a patient in the study.  As per the PLOS ONE policy ([http://journals.plos.org/plosone/s/submission-guidelines#loc-human-subjects-research](https://protect-au.mimecast.com/s/hWTiC6XQ4LfonyLj5fL-kbT?domain=journals.plos.org)) on papers that include identifying, or potentially identifying, information, the individual(s) or parent(s)/guardian(s) must be informed of the terms of the PLOS open-access (CC-BY) license and provide specific permission for publication of these details under the terms of this license. Please download the Consent Form for Publication in a PLOS Journal ([http://journals.plos.org/plosone/s/file?id=8ce6/plos-consent-form-english.pdf](https://protect-au.mimecast.com/s/VRvYC71R2NTmOZG4DU0tTgf?domain=journals.plos.org)). The signed consent form should not be submitted with the manuscript, but should be securely filed in the individual's case notes. Please amend the methods section and ethics statement of the manuscript to explicitly state that the patient/participant has provided consent for publication: “The individual in this manuscript has given written informed consent (as outlined in PLOS consent form) to publish these case details”. | Thank you - this has been amended |
| If you are unable to obtain consent from the subject of the photograph, you will need to remove the figure and any other textual identifying information or case descriptions for this individual. | Not applicable |
| 4. We note that the grant information you provided in the ‘Funding Information’ and ‘Financial Disclosure’ sections do not match. | Thank you - This has been amended |
| When you resubmit, please ensure that you provide the correct grant numbers for the awards you received for your study in the ‘Funding Information’ section. | Thank you - This has been amended – Marie Bashier Institute – grant # RC3241 PC RY859 |
| 5. Please ensure that you include a title page within your main document. You should list all authors and all affiliations as per our author instructions and clearly indicate the corresponding author. | Thank you - This has been amended, the title page is now added to the main manuscript and is formatted as per PLOS ONE's style requirements |
| 6. Please amend your manuscript to include your abstract after the title page. | Thank you – this has been amended the title page and abstract are now included with the manuscript |
| 7. Please include your table as part of your main manuscript and remove the individual files. Please note that supplementary tables (should remain/ be uploaded) as separate "supporting information" files. | Thank you - This has been corrected – figures and supporting information are now submitted as separate files and supporting information is listed at the end of the manuscript |
| 8. Please include captions for your Supporting Information files at the end of your manuscript, and update any in-text citations to match accordingly. Please see our Supporting Information guidelines for more information: [http://journals.plos.org/plosone/s/supporting-information](https://protect-au.mimecast.com/s/f9rlC81V0PTj7YNDoUR0Qvn?domain=journals.plos.org). | Thank you – this has been amended |
| Reviewers' comments: Reviewer's Responses to Questions |  |
| **Comments to the Author** 1. Is the manuscript technically sound, and do the data support the conclusions? The manuscript must describe a technically sound piece of scientific research with data that supports the conclusions. Experiments must have been conducted rigorously, with appropriate controls, replication, and sample sizes. The conclusions must be drawn appropriately based on the data presented.  Reviewer #1: Partly  Reviewer #2: Yes | Thank you. |
| 2. Has the statistical analysis been performed appropriately and rigorously?   Reviewer #1: Yes  Reviewer #2: N/A | Thank you. |
| 3. Have the authors made all data underlying the findings in their manuscript fully available?  The [PLOS Data policy](https://protect-au.mimecast.com/s/BUSaC91WPRTm524LGUGVZMr?domain=plosone.org) requires authors to make all data underlying the findings described in their manuscript fully available without restriction, with rare exception (please refer to the Data Availability Statement in the manuscript PDF file). The data should be provided as part of the manuscript or its supporting information, or deposited to a public repository. For example, in addition to summary statistics, the data points behind means, medians and variance measures should be available. If there are restrictions on publicly sharing data—e.g. participant privacy or use of data from a third party—those must be specified.   Reviewer #1: Yes  Reviewer #2: Yes | Thank you. |
| 4. Is the manuscript presented in an intelligible fashion and written in standard English?  PLOS ONE does not copyedit accepted manuscripts, so the language in submitted articles must be clear, correct, and unambiguous. Any typographical or grammatical errors should be corrected at revision, so please note any specific errors here.  Reviewer #1: Yes  Reviewer #2: No | Thank you. |
| 5. Review Comments to the Author  Please use the space provided to explain your answers to the questions above. You may also include additional comments for the author, including concerns about dual publication, research ethics, or publication ethics. (Please upload your review as an attachment if it exceeds 20,000 characters) |  |
|  |  |
| Reviewer #1: > The article addresses a key important topic, yet the conducted research and the outcome are really shallow. > Major concerns:  > - Only 8 patients participated in the study, and even the question guide was modified during the study period. This work is rather a pilot than real research. It can be a good first step to start a survey with 8 in-depth interviews to highlight critical points on acceptance of video monitoring. But the work should be continued; e.g. a short questionnaire should be designed that contains questions about the previously identified critical point.  A conclusion cannot be drawn from the answers of 8 patients.  > - The recruitment was via email. The paper does not mention how many emails were sent out. If much more than 8 then the probability of selection bias is quite large.  > - The title claims that the survey was about using video monitoring to measure hand hygiene compliance. In the later part, patients start to talk about the advantages of video monitoring in general. They would accept the use of the system because it can monitoring of falls; facilitate family communication; can be useful when a patients present sign of abuse; or when the patient can be abusive because of drugs, the recording can be a piece of evidence, it can prevent theft or stop healthcare-workers to do something that they should not do. As patients conclude, the acceptance of the system depends on whether the benefits outweigh the risks. If the paper summarizes ALL benefits that a video-monitoring system can provide, then it cannot be concluded that "video monitoring system for auditing hand hygiene broadly accepted". In that case, the right conclusion would be that this theoretical super-video system is broadly accepted.  > Minor remarks: > - At the abstract, before the 'Conclusion' part there is an empty row. > - The abstract uses the abbreviation VMS without explaining it  > - The words like "yeah", "well", "um...", "a...", "you know" can be omitted from the quotes.  > - In Figure 1, the gray and orange are quite similar when it is black&white printed. | We respectfully disagree. To our knowledge this is the first paper to report on health consumers perceptions in response to the trial of electronic monitoring of HCW hand hygiene behaviour.  We respectfully disagree and categorically reject the assertion that this was “not real research”. It is research that is subject to HREC approval and is funded.  The 8 participants represented a convenience sample and not an experimental study that would require statistically powered samples.  As is stated in the manuscript this work is part of a wider program of research and it is continuing.  We respectfully disagree with the statement that “A conclusion cannot be drawn from the answers of 8 patients” as it demonstrates the reviewer’s a lack of understanding of the study design and its suitability for the aim of the study.  Participants were a purposeful convenience sample, all participants who expressed interest were interviewed, and this was not an experimental study that would require the considerations regarding the management of sampling bias. Participants were health care consumers/patients recruited via a health care consumer foundation and associated networks. As is typical in qualitative studies they were recruited on the basis that they could help the researchers understand the problem being investigated via the provision of rich information.  We respectfully disagree. These individuals participated in an exploration of their responses to the trial of the technology – they were not independently surveyed or interviewed in an aloof manner.  Thank you – an explanation of the abbreviation VMS has been added to the abstract and the additional row has been removed  Thank you – we respectfully disagree - The participant’s quotations are reported verbatim and the use of pauses, “um”, “ahh” etc demonstrate the consideration participants gave to the questions and provide context and emotional tone.  Thank you – this has been ammended |
| Reviewer #2: Dear authors,  Greetings!  Although the manuscript is well documented it needs some clarification and corrections. Please specify the design: what do you mean by "pragmatic naturalistic framework"? | Thank you – this has been amended |
